# Survival benefit of platinum-based regimen in early stage triple negative breast cancer: A meta-analysis of randomized controlled trials

**DOI:** 10.1038/s41523-021-00367-w

**Published:** 2021-12-21

**Authors:** Lei Bian, Ping Yu, Jiahuai Wen, Na Li, Wanwei Huang, Xiaoming Xie, Feng Ye

**Affiliations:** 1 Department of Breast Oncology, Sun Yat-Sen University Cancer Center, State Key Laboratory of Oncology in South China, Collaborative Innovation Center for Cancer Medicine, Guangzhou, Guangdong China; 2 Department of Anesthesiology, Sun Yat-Sen University Cancer Center, State Key Laboratory of Oncology in South China, Collaborative Innovation Center for Cancer Medicine, Guangzhou, Guangdong China; 3 Department of Breast Oncology, Guangdong Hospital of Traditional Chinese Medicine, Guangzhou, Guangdong China

**Keywords:** Breast cancer, Outcomes research

## Abstract

Platinum (Pt)-based chemo-regimens have been proved effective in neoadjuvant and salvage chemotherapy of triple negative breast cancer (TNBC). However, the survival benefit of Pt-based regimens in early stage TNBC(eTNBC) treatment has remained unclear. We conducted a meta-analysis to explore its role in improving the clinical outcomes of eTNBC. We carried out a comprehensive literature search on 15 March 2021 for randomized controlled trials (RCTs) comparing ajuvant/neoadjuvant Pt-based and Pt-free chemo-regimens in eTNBC patients, according to PRISMA 2020. We extracted the survival data and utilized the STATA software to calculate the summarized hazard ratios (HRs) and 95% confidence interval (95% CI) for overall survival (OS) and disease-free survival (DFS). Seven eligible RCTs enrolling a total of 2,027 eTNBC patients were identified in this meta-analysis, with 1,007 receiving Pt-free regimens, and the other 1,020 patients receiving Pt-based regimens, respectively. Patients in Pt-based regimens arm were associated with significant improved DFS (HR = 0.70, 95% CI: 0.58–0.84), and OS (HR = 0.78, 95% CI: 0.61–1.00). The survival benefits of DFS remained consistent in both the two strategies of Pt usage, either adding Pt to standard anthracyclines&taxanes based regimens (A&T + Pt), or combination of Pt and taxanes alone (TPt). The survival benefits also remained consistent in either neoadjuvant or adjuvant use of Pt. The present meta-analysis of RCTs revealed that Pt-based chemo-regimens could significantly improve both DFS and OS for eTNBC patients. Based on efficiency and toxicity, we recommend Pt-based regimens for eTNBC, especially the “A&T + Pt” mode if the toxicities are tolerable, which may lead TNBC therapy into a new era.

## Introduction

Triple negative breast cancer (TNBC), i.e., ER (estrogen receptor)-, PR (progesterone receptor)-, and HER-2 (human epithelial growth factor receptor-2)-, accounting for 15−20% in breast cancer, is a highly aggressive subtype with a significantly inferior prognosis than non-TNBC^1^. Due to insensitivity to endocrine therapy and anti-HER2 therapy, chemotherapy is the dominant systemic treatment for TNBC in general. To date, anthracyclines (A/E) and taxanes(T) based chemo-regimens, administrated in various combinations and schedules, have been widely accepted as the standard regimens for early stage TNBC (eTNBC)^2,3^. In the past decade, dose-dense regimens have been proved to be more effective in adjuvant treatments of high-risk BC patients^4,5^. Moreover, neoadjuvant chemotherapy (NACT) has also provided more information in tailoring subsequent treatments for BC patients. Adjuvant addition of capecitabine for those TNBC patients with non-pCR (pathological complete response) after standard NACT has been recommended in most guidelines^6^. However, even with the assistance of dose-dense regimens and guidance of NACT, the improvement of long-term survival is still critical for eTNBC.

Platinum (Pt) agents, usually referring to carboplatin (Cb) and cisplatin(DDP), are cytotoxic DNA-damaging chemo-drugs widely used in various malignancies. Platinum can cause DNA strand breaks, consequently leading to cell apoptosis^7^. Thus platinum is believed to be specially active in those cancer cells with DNA repair deficiency. Since TNBC has been reported to harbor more proportion (>50%) of homologous DNA recombination defects, either due to BRCA mutation or other mechanisms, it’s supposed that TNBC might be more sensitive to Pt agents^8–10^. A latest meta-analysis of randomized controlled clinical trials (RCTs) from Cochrane library in 2020 indicated that Pt-containing regimens might improve time to progression or progression-free survival (TTP/PFS) of metastatic TNBC (mTNBC) patients, and provide a small benefit to overall survival (OS)^11^. These data, along with other real-world studies^12^, support the role of Pt in the first-line salvage chemotherapy for mTNBC.

On the other hand, for eTNBC, many studies explored the value of Pt in the neoadjuvant phase, and the most common method was to add Pt to standard A&T-based regimens (A&T + Pt)^13–15^. Results of these studies demonstrated that “A&T + Pt” significantly increased pCR rates and the risk of grade 3/4 hematological adverse events (AEs) in eTNBC, when compared with A&T alone^16,17^.

Despite efficiency in salvage and neoadjuvant therapy of TNBC, the survival benefits of Pt-based regimens in eTNBC remain unclear^17^. Some retrospective or single-armed studies indicated that long term survival of eTNBC in Pt-based groups was non-inferior to standard A&T-based regimens^8,17–19^. However, results from properly designed RCTs were inadequate. Many RCTs evaluated Pt-based regimens in eTNBC patients, with most of them concentrating on the pCR rate in neoadjuvant therapy. Few studies reported the long-term clinical outcomes and the results were controversial^16^. Thus no agreement has been reached on the indications of platinum use in eTNBC so far, and even in the neoadjuvant phase, the debate continues^20^.

Recently, several RCTs evaluated the value of Pt-based regimens in adjuvant treatments of eTNBC. PATTERN trial reported that 6 cycles of “TPt” regimen(paclitaxel plus carboplatin) gained a better survival in eTNBC, compared with a standard-dose regimen of 3FEC (fluorouracil, epirubicin, and cyclophosphamide) -3T (docetaxel)^21^. Similarly, Du et al. demonstrated that six cycles of TPt regimen was non-inferior to a standard 4EC-4T regimen^22^. With increasing and conflicting results reported by different trials, we conducted the present meta-analysis of RCTs to evaluate the controversial value on the survival of eTNBC for Pt-based regimens.

## Results

### Eligible studies

**Fig. 1:**
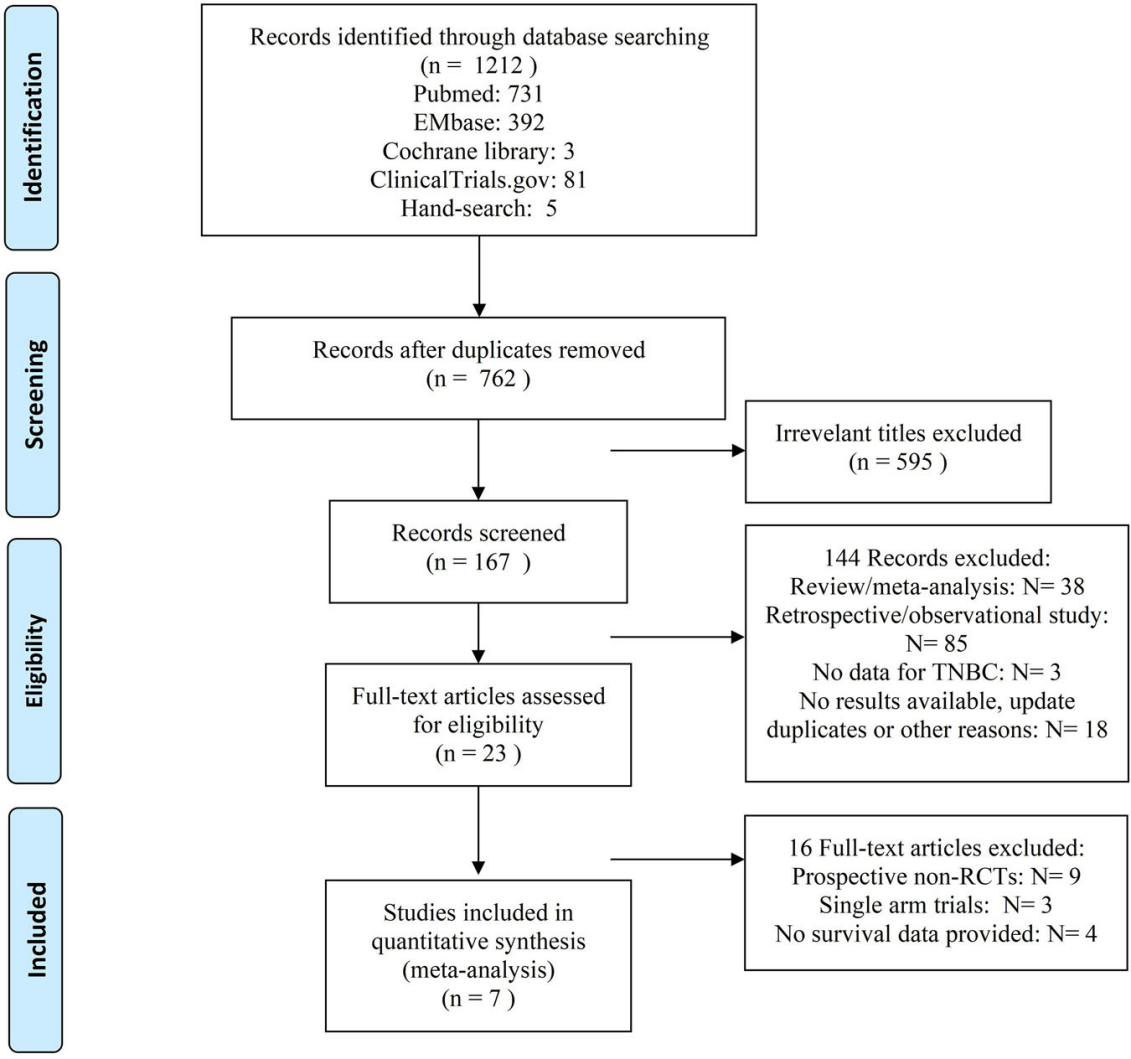
The PRISMA flow diagram.

The systematic search of databases (EMbase, Pubmed, the Cochrane library, Clinical Trials.gov) and international conferences yielded one thousand and two hundred and twelve publications (1,212) in all. Seven hundred and sixty-two articles (762) were left after excluding duplication during the first screening. After title and abstract revision, another 595 articles were excluded. During full-text accession, 144 articles and further 16 articles were excluded with reasons. In the end, seven RCTs that met the eligibility criteria, were included^21–27^. The PRISMA flow diagram is shown in Fig. 1.

### Characteristics of enrolled trials

Ultimately, seven eligible RCTs enrolling a total of 2,027 eTNBC patients were identified in this meta-analysis, with 1,007 receiving Pt-free regimens, and the other 1,020 patients receiving Pt-based regimens, respectively. All eligible trials have published articles with full text. There were two strategies of Pt usage in Pt-based regimens: either adding Pt to standard A&T-based regimens (A&T + Pt, 4trials), or combined use of Pt and taxanes alone (TPt, 3 trials). As to the stage of therapy, 4 trials applied chemotherapy in neoadjuvant phase, while the other 3 trials in adjuvant phase. The median follow-up varied from 39 to 79 months. DFS and OS data could be extracted in all six trials. The characteristics and survival data of the eligible studies were presented in Table 1.

**Table 1:** Characteristics of eligible studies.

Study	Latest update	Type of trial (registry number)	Enrollment period	Age range (year)	Chemo-regimen-Control arm-Platinum arm	TNBC NO.-Control-Platinum	Carboplatin dosage	TNM stage	Stage of therapy	Median follow-up	HR (95% CI)	
											DFS	OS
CALGB 40603	2015	RCT(NCT00861705)	2009.5−2012.8	18–70	−P/PB*12w-4ddAC−P/PBCb*12w-4ddAC	−212−221	AUC 6, d1, q3w	Stage II/III	Neoadjuvant	39m	0.84 (0.58−1.22)	1.15 (0.74–1.79)
Khalid	2015	RCT(NS)	2008.1−2014.12	17–65	−3CEF-3T−3CEF-3TCb-1yCM	−80−78	AUC 5, d1, q3w	Stage II/III;*T* > 1 cm/LN+	Adjuvant	52m	0.61 (0.39–0.96)	0.56 (0.32–0.98)
Zhang	2016	RCT(NS)	2006.5−2012.12	24–73	−4-6EP−4-6PCb	−44−47	AUC 5, d1, q3w	Stage II/III	Neoadjuvant	55m	0.56 (0.25–1.27)	0.70 (0.22–2.26)
GeparSixto	2018	RCT(NCT01426880)	2011.8−2012.12	48	−PM*18w+Beva−PMCb*18w+Beva	−157−158	AUC 1.5/2, d1, qw	Stage II/III	Neoadjuvant	47.3m	0.56 (0.34–0.93)	0.60 (0.32–1.12)
PATTERN	2020	RCT(NCT01216111)	2011.6−2016.4	18–70	−3CEF-3T-6PCb	−322−325	AUC 2, d1, 8, 15, q28d	N_1-3_, or T_1c-4a_N_0_, M_0_	Adjuvant	62m	0.65 (0.44–0.96)	0.71 (0.42–1.22)
Du	2020	RCT(NS)	2009.7−2015.10	48	−4EC-4T-6TCb	−154−154	AUC 5, d1, q3w	N_1-3_, or T_1c-4a_N_0_, M_0_	Adjuvant	66.9m	1.11 (0.65–1.89)	1.27 (0.49–3.32)
Iwase	2020	RCT(UMIN000003355)	2010.3−2011.9	47 (30–70)	−3T-3CEF-3TCb-3CEF	−38−37	AUC 5, d1, q3w	Stage II/III A	Neoadjuvant	6.6y	0.22 (0.06–0.82)	0.12 (0.01–0.96)

Definition of TNBC: in CALGB 40603: (ER/PR < 10%+, HER2−); in other eligible studies: (ER/PR−, HER2−).

When age range was not shown, the median age is filled in.

Abbreviations: *RCT* randomized controlled trials, *TNBC* triple-negative breast cancer, *P/T* paclitaxel/docetaxel, *E* epirubicin, *C* cyclophosphamide/CTX, *F* 5-fuorouracil, *A* anthracycline, *Cb* carboplatin, *1yCM* oral metronomic chemotherapy of (CTX + MTX) for 1 year, *PM* paclitaxel+nonpegylated liposomal doxorubicin (NPLD, MyocetVR), *B/Beva* bevacizumab. *No.* number.

### Efficacy of Pt-based regimens

**Fig. 2:**
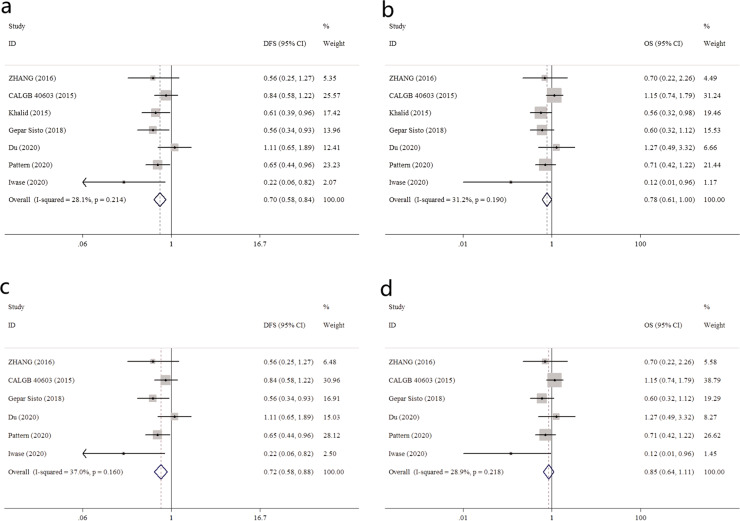
Overall efficiency for Pt-based regimen in early stage TNBC. **a** Summarized HR for DFS, all RCTs included; **b** summarized HR for OS, all RCTs included; **c** summarized HR for DFS, with (Khalid, 2015) trial excluded; **d** summarized HR for OS, with (Khalid, 2015) trial excluded.

As mentioned above, DFS and OS data of TNBC patients could be extracted in all seven RCTs (*N* = 2,027 eTNBC patients; Control arm: 1,007, Platinum arm: 1,020). For DFS, the separate and pooled HRs and 95% CIs were shown in Fig. 2a. No between-study heterogeneity was noted (*p* = 0.214, I-square = 28.1%). The summarized estimate HR of the Platinum arm versus the Control arm was 0.70 (95% CI: 0.58–0.84). The summarized HR for OS was shown in Fig. 2b. Utilizing a fixed-effects model, a better OS was also indicated for Platinum arm: pooled HR = 0.78 (95% CI: 0.61–1.00), with no significant heterogeneity (*p* = 0.19, I-square = 31.2%).

The summarized HRs above indicated that Platinum arm had both significantly superior DFS and OS, when compared with Control arm. However, as presented in Table 1, one trial (Khalid, 2015) was a bit different: the Pt-based regimen did not only add platinum (Carboplatin) alone, but also 1 year oral metronomic chemotherapy (CTX + MTX). To reduce bias, we repeated the analysis in the left six trials (*N* = 1,869 eTNBC patients; Control arm: 927, Platinum arm: 942). The survival benefits of platinum in eTNBC were similar: for DFS, pooled HR = 0.72 (0.58–0.88); for OS, pooled HR = 0.85 (0.64–1.11); both without significant heterogeneity, as shown in Fig. 2c, d.

Given the two strategies of Pt-based regimens: “A&T + Pt” mode, or “TPt” mode, we further conducted sub-analysis.

**Fig. 3:**
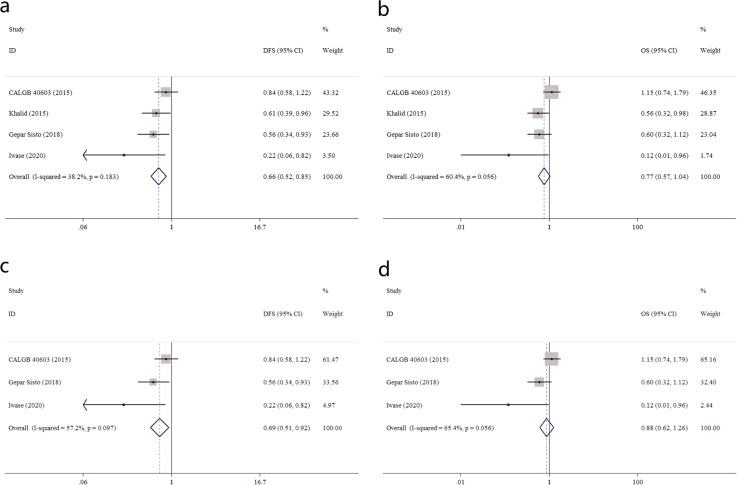
Subgroup analysis in eTNBC patients receiving “A&T + Pt” mode regimen. **a** Summarized HR for DFS; **b** summarized HR for OS; **c** summarized HR for DFS, with (Khalid, 2015) trial excluded; **d** Summarized HR for OS, with (Khalid, 2015) trial excluded.

Four trials applied the “A&T + Pt” mode (*N* = 981 eTNBC patients; Control arm: 487, Platinum arm: 494). As shown in Fig. 3a, b, a significantly better DFS outcome was indicated in the Platinum arm (HR = 0.66, 0.52–0.85), while the Platinum arm trend towards a better OS (HR = 0.77, 0.57–1.04). Similarly, if we excluded Khalid trial, the pooled HR for DFS was 0.69 (0.51–0.92), while for OS was 0.88 (0.62–1.26), shown in Fig. 3c, d.

**Fig. 4:**
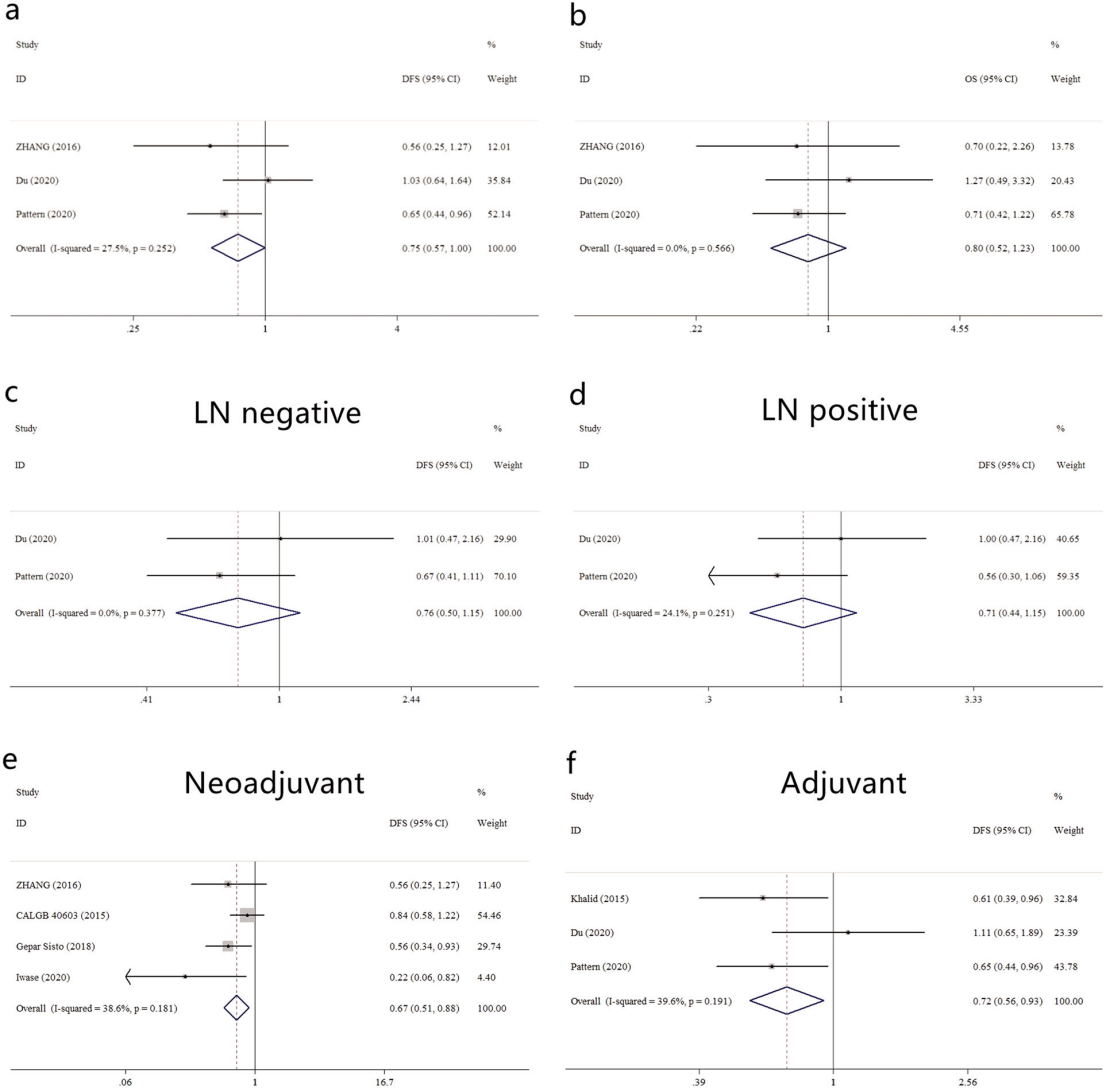
Subgroup analysis in eTNBC patients. **a** Summarized HR for DFS in eTNBC patients receiving “TPt” regimen; **b** summarized HR for OS in eTNBC patients receiving “TPt” regimen; **c** summarized HR for DFS in LN negative eTNBC patients; **d** summarized HR for OS in LN positive eTNBC patients; **e** summarized HR for DFS in eTNBC receiving neoadjuvant Pt-based regimen; **f** summarized HR for OS in eTNBC receiving adjuvant Pt-based regimen.

Three trials applied the “TPt” mode (*N* = 1,046 eTNBC patients; Control arm: 520, Platinum arm: 526). As shown in Fig. 4a, b, a trend towards better DFS was indicated in the Platinum arm (HR = 0.75, 0.56–1.01), while pooled HR for OS in the Platinum arm was 0.80 (0.52–1.23). Two of the three trials(PATTERN and Du) provided the subgroup analysis according to LN status. As shown in Fig. 4c, d, pooled HR for DFS in the LN negative patients was 0.76 (0.50–1.15), while in the LN positive patients was 0.71 (0.44–1.15).

Finally we conducted the analysis according to the stage of therapy (neoadjuvant/adjuvant). As shown in Fig. 4e, f, for 4 neoadjuvant RCTs (Zhang, CALGB 40603, GeparSixto and Iwase), pooled HR for DFS was 0.67 (0.51–0.88). For the other 3 adjuvant RCTs, pooled HR for DFS was 0.72 (0.56–0.93).

### Toxicity

In previous meta-analysis^17^, the safety of “A&T + Pt” mode in eTNBC had been evaluated. Compared with “A&T”, the risk of grade 3/4 AEs was significantly raised for “A&T + Pt” mode. In the present study, we concentrated on the safety of “TPt” mode regimen in eTNBC treatments. We analyzed data from the three trials (Zhang 2016, Du 2020, PATTERN 2020), in which TCb regimens were applied in the Pt-based arm. The safety profiles of TCb regimens and the control group(the Pt-free “A&T” based regimens) are listed in Table 2. Compared with “A&T” based regimens, TCb regimen showed decreased risk for most AEs in general. As to Grade 3/4 Haematologic AEs, TCb regimen caused less Neutropaenia, Leukopenia, but more Anaemia and Thrombocytopaenia. For non-hematological toxicity, AEs in patients receiving TCb regimen were generally less than “A&T” based regimens, especially less Grade 3/4 Anorexia/Nausea, Diarrhea/Abdominal pain, Alopecia, although most non-hematological AEs in the two arms were tolerable.

**Table 2:** Summarized toxicities data for TCb regimens.

Adverse events	AEs No./No. (%)		*p* value
	TCb group	A&T group	
*Haematologic toxicity (Grade 3/4)*			
Neutropaenia	384/523 (73.4)	425/518 (82.0)	<0.001**
Febrile neutropaenia	3/322 (0.9)	30/320 (9.4)	<0.001**
Leukopenia	315/476 (66.2)	371/474 (78.3)	<0.001**
Anaemia	35/476 (7.4)	7/474 (1.5)	<0.001**
Thrombocytopaenia	26/523 (5.0)	10/518 (1.9)	0.007**
*Non-hematological toxicity*			
Anorexia/Nausea (Grade 3/4)	7/476 (1.5)	16/474 (3.8)	0.05*
Vomiting (Grade 3/4)	4/369 (1.1)	10/364 (2.7)	0.10
Diarrhea/Abdominal pain (Grade 3/4)	5/322 (1.6)	13/320 (4.1)	0.05*
Alopecia (Grade 3/4)	27/154 (17.5)	56/154 (36.4)	<0.001**
ALT/AST elevation	74/523 (14.1)	75/518 (14.5)	0.879
Peripheral neuropathy	46/201 (22.9)	62/198 (31.3)	0.06
Fatigue	17/154 (11.0)	21/154 (13.6)	0.488
Myalgia/arthralgia	17/201 (8.5)	28/198 (14.1)	0.07
ST-T changes in ECG	35/201 (17.4)	47/198 (23.7)	0.11

*NS* not shown.

### Publication bias

**Fig. 5:**
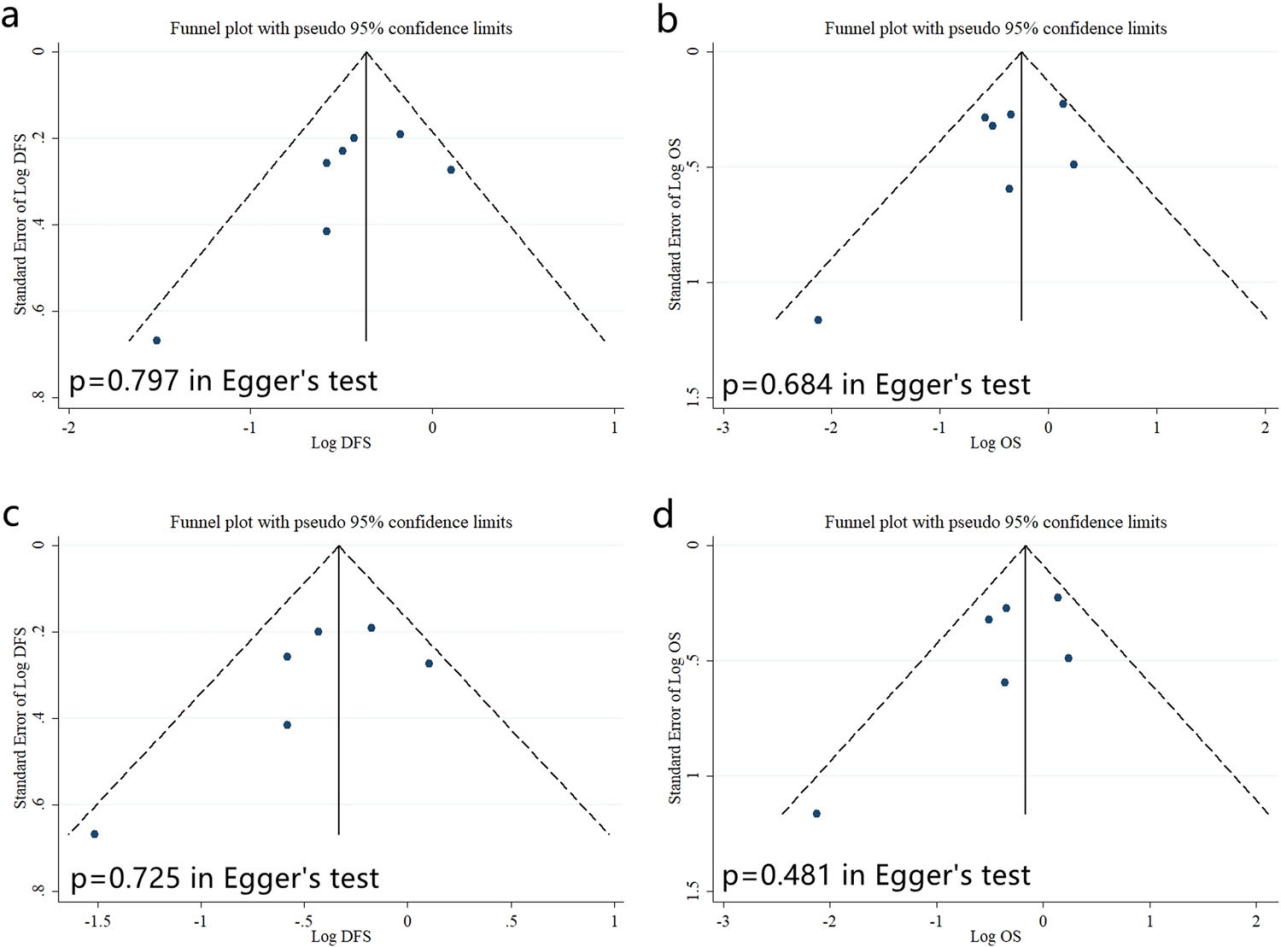
Funnel plots and Egger’s test evaluating the publication bias. No significant publication bias identified in the data pooling (Funnel plot in Fig. 5a–d corresponding to Fig. 2a–d).

Egger’s test and funnel plot are used to detect and describe the publication bias. As shown in Fig. 5a–d, there were no significant publication bias identified in the data pooling (Funnel plot in Fig. 5a–d corresponding to Fig. 2a–d).

## Discussion

How to improve clinical outcomes of early stage TNBC is still a critical issue^28^. Despite the great progress in genotype profiles of TNBC^29^ and immune check point (PD-1/PD-L1 antibodies) therapy^30^, the most acknowledged change in eTNBC treatments was the NACT-guided mode. The CREATE-X trial demonstrated that adjuvant capecitabine could significantly ameliorate the prognosis in those eTNBC with non-pCR after standard NACT. Thus, indications for NACT have been extended to eTNBC patients with risk factors (i.e., *T* > 2 cm, or LN+, or young-onset (<40 y), or high ki67 index). A latest meta-analysis evaluated the value of pCR after NACT in breast cancer according to different subtypes and found that the associations between pCR and good DFS were most definite in TNBC(pCR vs non-pCR, HR = 0.18, 95% CI: 0.10–0.31)^31^. Thus the authors recommended pCR rate to be a surrogate of DFS in eTNBC patients.

There have been various studies and trials exploring how to improve the pCR rate of NACT in eTNBC, and Pt-based regimens were among those efforts. Despite the significantly elevated pCR rate of TNBC receiving Pt-based regimens, the benefits in survival, i.e., DFS/RFS/OS, have not been reported in most previous trials and the few existing data are not consistent. Due to the toxicities and conflicting survival benefits of Pt-based regimens, the role of platinum in eTNBC treatments has not been widely accepted, even in the neoadjuvant therapy.

In this meta-analysis, we included several latest trials with survival data, such like Iwase 2020, Du 2020, and PATTERN trial, thus making this article more meaningful and convincing in respect of evaluating the survival benefits of Pt in eTNBC.

The present research proved that Pt-based regimens could bring survival benefits, especially a better DFS, to eTNBC patients, compared with Pt-free standard chemo-regimens. The results would definitely help clinicians in decision-making on the use of Pt.

Moreover, we explored the value of the two strategies of Pt-based regimens: the “A&T + Pt” mode and the “TPt” mode. The subgroup analysis confirmed the improved DFS of both two strategies in eTNBC patients, especially the “A&T + Pt” mode. In another hand, analysis according to adjuvant/neoadjuvant stages indicated that Pt-based regimens in both stages provided better DFS. These results gave strong supports to Pt use in neo-/adjuvant therapies in eTNBC patients.

During the peer review of this article, we also acknowledged that Loibl et al reported the survival data of an important trial, the BrighTNess trial^32^, at ESMO 2021. The HR for DFS with addition of carboplatin alone vs standard “A&T” regimen was 0.57 (95% CI: 0.36–0.91, *p* = 0.018), and that HR for OS was 0.63 (95% CI: 0.33–1.21, *p* = 0.166). These outcomes of BrighTNess trial were consistent with our study. To make our results more timely, we added the data of BrighTNess trial and calculated the summarized HRs for DFS and OS, shown in Supplementary Fig. 1. (NOTE: This was just a pool-up of data, not a rigorous meta-analysis after systemic literature search.) The overall efficiencies of Pt-based vs Pt-free regimens were shown in Supplementary Fig. 1A, B: pooled HR for DFS was 0.68 (0.57–0.81) and for OS was 0.76 (0.60–0.0.96), which were similar with those in Fig. 2a, b. And the efficiency of “A&T + Pt” mode was also similar but more definite (Supplementary Fig. 1C, D). Based on the existing data, we recommend the Pt-based regimens for eTNBC patients, especially the “A&T + Pt” mode if the toxicities are tolerable.

Our study has several limitations. Firstly, the seven RCTs included in this meta-analysis applied various A&T-based regimens as control arms and no trials used the dose-dense AC-T (ddAC-T) regimen, as recommended in most present international guidelines. Thus the superiority of Pt-based regimens could be weakened, when compared with ddAC-T in the actual clinical work nowadays.

Secondly, TNBC tends to harbor more germline gene mutations of BRCA1/2, Tp53 et al., accounting for 15–20% as reported. However, in this research, we did not conduct a sub-analysis according to BRCA1/2 status, since six of the seven trials did not provide the information.

Thirdly, TNBC has been proved to be malignancies with heterogeneity and could be classified into intrinsic molecular subtypes according to a series of genomic, transcriptomic, or metabolic landscapes in recent years^30,33,34^. However, information of intrinsic molecular subtypes of TNBC were deficient in the enrolled RCTs, and thus which group of TNBC would mostly benefit from Pt-based regimens remain unclear, too.

To sum up, Pt-based regimens can bring survival benefits to eTNBC patients based on the existing data. We are also expecting the upcoming data from important ongoing trials like NRG-BR003, to further confirm our conclusion. More RCTs are needed to elucidate their value in different TNBC subtypes in future.

In conclusion, the present meta-analysis of RCTs revealed that Pt-based chemo-regimens could significantly improve both DFS and OS for eTNBC patients. Based on efficiency and toxicity, we recommend Pt-based regimens for eTNBC, especially the “A&T + Pt” mode if the toxicities are tolerable, which may lead TNBC therapy into a new era.

## Methods

### Searching strategy and publication selection

We carried out a systemic literature search on 15 March, 2021 of online databases, including EMbase, Pubmed, the Cochrane library, and ClinicalTrials.gov, for all studies concerning on Pt-based regimens in early stage BC patients by two reviewers (FY and LB) independently. The searching strategy is to search the following words in the title/abstract: (breast cancer) AND (platinum OR carboplatin OR cisplatin) AND (neoadjuvant therapy OR pre-operative therapy OR adjuvant therapy). Only studies in English language were included.

We have also gone through the cross-referenced articles, and the potential relevant unpublished studies on important international conference websites, including the ESMO (European Society for Medical Oncology), ASCO (American Society of Clinical Oncology), and SABCS (San Antonio Breast Cancer Symposium).

This study has been registered at PROSPERO 2021 (ID: CRD42021243344, Available from: https://www.crd.york.ac.uk/prospero/display_record.php?ID=CRD42021243344).

### Ethics approval and consent to participate

Ethics approval and trial registration number are not applicable for the present meta-analysis.

### Inclusion and exclusion criteria

The inclusion criteria for eligible trials were: (1) prospective RCTs recruiting eTNBC patients(stage I-III), either as the whole cohort, or as a subgroup; (2) studies should include a comparison of survival between at least two arms of eTNBCs, with a Control arm receiving Pt-free standard neo-/adjuvant chemo-regimens and a Platinum arm receiving Pt-based chemo-regimens. Standard chemotherapy was defined as anthracyclines&taxanes(A&T) based regimens. Pt-based regimens could be either adding Pt to standard A&T based regimens (A&T + Pt), or a combination of Pt and taxanes alone (TPt); (3) detailed survival data of DFS/relapse-free survival (RFS) and OS, should be presented in the studies; (4) In case of duplicate trials, only the latest publication with complete data was included.

The exclusion criteria for trials were: (1) observational/retrospective studies or prospective Non-RCTs; (2) single-arm trials or studies without proper control groups; (3) studies without detailed survival data; (4) ongoing trials without reported results.

### Definitions of survival data

OS: date of surgery or diagnosis (referred to “date”) to death;

BCSS (breast cancer-specific survival): date to death due to breast cancer progression.

DFS: date to the first occurrence of any event (either in situ or invasive breast cancer), including local relapse (LRR, defined as the appearance of tumors in ipsilateral breast/chest wall, axillary, infra/supra-clavicular area, internal mammary area), distant metastasis (DM, defined as other recurrences except LRR), contralateral breast cancer, second primary cancer, or death from any cause.

RFS, LRRFS, and DMFS: date to LRR and/or DM, LRR and DM, or death from any cause, respectively.

### Data extraction and study objectives

Two independent investigators (FY and LB) performed the data extraction. The following information was extracted if available: author/trial name, latest update year, type of study (with registry number), enrollment period, age range, number (No.) of TNBC patients in each arm, chemotherapy regimens, platinum dosage, and usage, stage of therapy (adjuvant or neoadjuvant), clinical stage, median follow-up years, nodal and menopausal status, and BRCA1/2 germline status in TNBC patients if available. Survival data were extracted if available, otherwise, we extracted and transformed them from the survival curve.

The primary objective of the present meta-analysis was to evaluate the survival benefits of Pt-based regimens in eTNBC patients, compared with Pt-free standard regimens. The major indexes were DFS/RFS and OS. The secondary objective was to evaluate the toxicities of Pt-based regimens, and the according indexes were AEs.

### Statistical analysis

The HRs and the corresponding 95% CI for each survival data of the eligible studies were extracted and summarized. Chi-square test-based Q statistics and I2 test were utilized to detect heterogeneity among the studies. *p* < 0.05 /I-square > 50% indicated heterogeneity. In case of significant heterogeneity, a random-effect model was applied, otherwise, a fixed-effect model instead. Sum-up of the separate and pooled survival data, along with the weights of each study, were shown in forest plots.

We also applied funnel plots and Egger’s test (indicated by *p* < 0.05) to determine the publication bias. All statistical analysis was performed with Stata 12.0 software with two-sides *p* values.

### Reporting summary

Further information on research design is available in the Nature Research Reporting Summary linked to this article.

## Supplementary information

Supplementary file

Reporting Summary

## Data Availability

All data generated or analyzed during this study are included in published article (and its supplementary information files) as indicated.

## Code availability

The code for calculating pooled HRs is written in STATA 12.0 software and available upon request.
