# Supplementary material for: Survival benefit of platinum-based regimen in early stage triple negative breast cancer: A meta-analysis of randomized controlled trials
Source: NPJ Breast Cancer. 2021 Dec 21;7:157. doi: 10.1038/s41523-021-00367-w (PMC8692362; doi:10.1038/s41523-021-00367-w)

**Supplementary Figure 1**. Efficiency for Pt-based regimen in early stage TNBC with addition of BrighTNess trial.

(**1**A) summarized HR for DFS;

(**1**B) summarized HR for OS;

(**1**C) summarized HR for DFS in patients receiving "A&T + Pt" mode regimen;

(**1**D) summarized HR for OS in patients receiving "A&T + Pt" mode regimen.

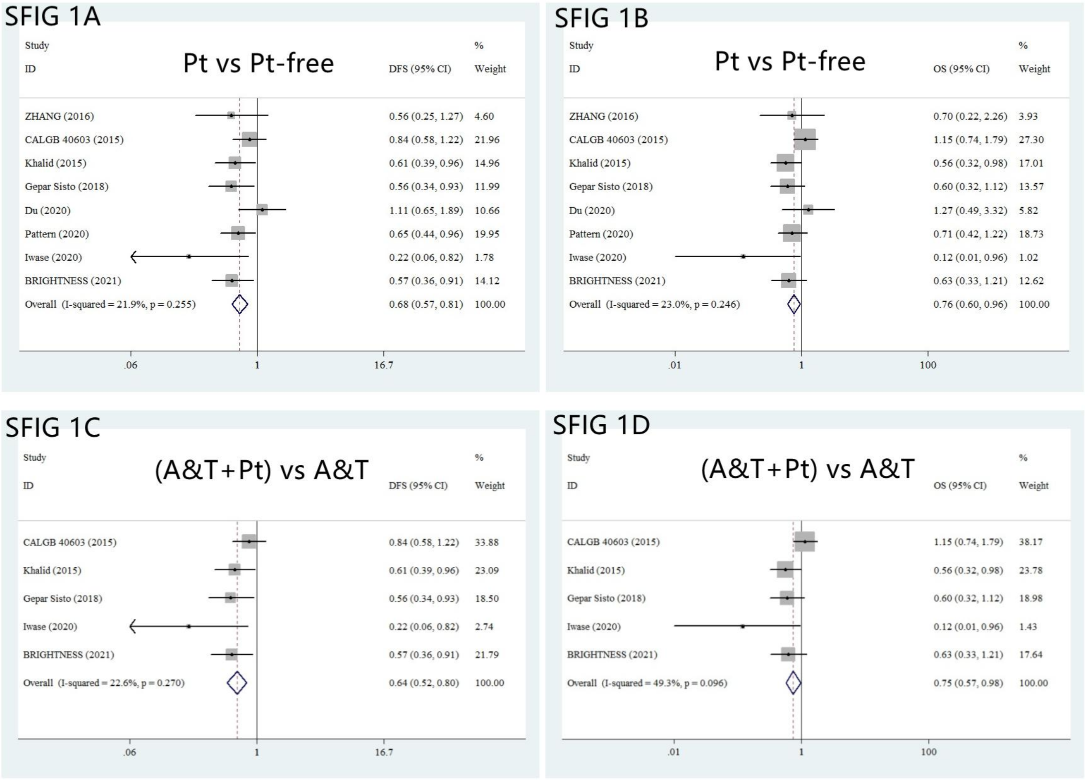

Supplement: Supplementary file 1 — Supplementary file [file 41523_2021_367_MOESM1_ESM.pdf]
